# AI, libraries, and literacy: revisiting the *Journal of the Medical Library Association*'s policy on the use of generative artificial intelligence in submissions

**DOI:** 10.5195/jmla.2026.2516

**Published:** 2026-10-01

**Authors:** Alexander J. Carroll, Jill T. Boruff, Michelle Kraft

**Affiliations:** 1 alexander.j.carroll@vanderbilt.edu, Associate Editor, Journal of the Medical Library Association, Associate Director, Stevenson Science and Engineering Library, Vanderbilt University, Nashville, TN, United States; 2 jill.boruff@mcgill.ca, Co-Lead Editor, Journal of the Medical Library Association, Associate Librarian, Schulich Library of Physical Sciences, Life Sciences, and Engineering, McGill University, Montreal, QC, Canada; 3 kraftm@ccf.org, Co-Lead Editor, Journal of the Medical Library Association, Medical Library Director, Cleveland Clinic Foundation, Cleveland, OH, United States

**Keywords:** Artificial Intelligence, information literacy, AI in Libraries, AI literacy, literacy skills, lifelong learning, scholarly communication, scholarly publishing, scholarly writing

## Abstract

In the fall of 2023, the *Journal of the Medical Library Association* (*JMLA*) introduced its policy on generative artificial intelligence (AI) submissions, predicting that these tools would become embedded within the workflows of clinicians, educators, researchers, and students. Those predictions dramatically underestimated AI's impact. On the three-year anniversary of that editorial, we revisit the issue in light of AI's unprecedentedly rapid adoption across knowledge work. We examine the educational challenges AI poses, arguing that excessive cognitive offloading threatens the deep thinking on which learning depends. We then consider the challenges AI creates for researchers, from hallucinated citations to fabricated diseases. We suggest that health sciences librarians, who offer expertise in approaching information systems with discernment, are well suited to be leaders in this space. To help prospective *JMLA* authors weigh whether and when to use AI, we propose the Compliance, Verifiability, and Disclosure (CVD) Framework. Throughout, we reaffirm our conviction that education and research must remain human-centered endeavors.

## INTRODUCTION

In the fall of 2023, the *Journal of the Medical Library Association* introduced its policy on generative artificial intelligence (AI) submissions. In our editorial introducing this policy, we predicted that AI applications would become embedded into the workflows of clinicians, educators, researchers, and students, which would in turn affect health sciences librarianship [1]. Although our policy advocated against unrestricted use of AI tools during manuscript preparation, we recognized the transformative potential of these tools and encouraged the *JMLA* community to explore using them.

Our predictions dramatically underestimated AI’s impact [2, 3]. The speed of these tools adoption is unprecedented [4]; by December 2024, ChatGPT users sent more than one billion messages each day, just over two years after its public release[5]. By comparison, it took Google nearly eight years to reach this same one billion daily uses benchmark [6]. In July 2025, over 750 million accounts were using ChatGPT each week, which represents nearly 10% of the global adult population [7]. Rogers’ diffusion of innovation theory suggests that AI usage would be concentrated among a handful of early adopters [8], yet these tools are now being used widely among different demographic groups across the world [9]. This widespread integration of AI tools runs contra to early forecasts of how AI adoption would manifest among workers generally and librarians in particular [10, 11].

However, adoption of AI tools in the workplace is not only a reflection of individual workers’ preferences. Businesses across many industries, including healthcare, are partnering with vendors to embed AI within their own networks. Firms investing in AI infrastructure are increasingly expecting their workers to demonstrate how they are using AI, with some technology firms evaluating individual worker productivity based on their quantity of AI tool usage. For those of us familiar with Goodhart’s Law, this has led to the predictable result of workers performatively using AI in order to appear more productive [12, 13]. Although affirmative adoption of AI remains contested within librarianship [14, 15], AI applications have become so thoroughly integrated into enterprise software that refusal to use AI is no longer tenable for many information professionals [16]. These tools’ rapid ascension has prompted us to revisit generative AI on the three-year anniversary of our previous editorial on AI.

## EDUCATIONAL CHALLENGES CREATED BY AI TOOLS

The most advanced AI models appear superintelligent, responding accurately and rapidly to complex questions designed to assess clinician reasoning skills [17]. Although the most robust models often require subscription fees to access, even free versions of AI tools can interpret and answer quantitative and qualitative questions alike [18].

Yet the ubiquity of AI has proven double-edged: AI’s presence within workplaces and classrooms invites workers and students to become overly reliant upon it [19]. Early evidence on the integration of AI into workplaces and educational settings suggests that using these tools does not facilitate permanent skill development [20], and in fact may inhibit learning new skills [21].

That AI tools could inhibit learning follows from our understanding of how humans learn: deep thinking is essential for learning new things [22]. Repeated exposure to concepts and intentional practice applying skills improves retention and transfer [23], which are essential for promoting lifelong learning [24]. However, thinking is a cognitively intensive task that can be psychologically unpleasant in the short term, so learners often seek to avoid cognitively challenging tasks when possible. Educators account for this by designing “desirable difficulties,” assignments that push students to persevere past these difficult thresholds and to achieve real learning [25]. AI tools can disrupt this model of learning, as they present a ready means for students to solve assignments that instructors use to create desirable difficulty.

AI tools’ capacity to trivialize major assignments has led to concerns that AI will promote maladaptive, excessive cognitive offloading behaviors among students. Excessive use of these tools within classroom environments may limit students’ ability to internalize knowledge and to think critically [26]. Excessive cognitive offloading is most likely to occur when novices are allowed to use AI tools without guardrails. Large language models have a handful of critical limitations baked into their probabilistic design that ensure that they can never be unfailingly correct in all instances [27]. However, novices learning about a new topic lack the prior knowledge needed to recognize when an AI is offering mistakes in facts or interpretations [28]. This portends a vicious cycle: students who become reliant upon AI will fail to gain the lasting, broad-based knowledge of the world needed for critical thinking, which will in turn push the learners to use AI for even more tasks.

Yet cognitive offloading is not inherently maladaptive [29]. Because the human’s brain capacity to store information within long-term, short-term, and working memory is limited [30], without cognitive offloading, humans’ ability to perform analytical reasoning is limited by the knowledge they can store in their mind at one time [31]. The development of literacy enabled humans to write down and physically store knowledge elsewhere until it was needed, leading to the creation of libraries and librarianship [32]. The human brain’s ability to use literacy to offload and onramp knowledge is why libraries eventually became essential infrastructure for modern health care and the scientific research enterprise [27]. As curators of cognitively offloaded knowledge, librarians have a clear role to play in guiding novices’ use of AI tools.

## RESEARCH CHALLENGES CREATED BY AI TOOLS

While the term “novices” often connotes students, researchers publishing outside of their narrow band of expertise can cognitively behave like novices. The downstream effects of researchers using AI tools to publish outside their areas of expertise are no longer hypothetical. Hallucinated citations to non-existent papers are now a common occurrence within published papers [33]. AI tools have been linked to a massive increase in low-quality manuscripts [34], a trend so intense within computer science that the preprint server arXiv suspended accepting review article submissions in early 2026 [35]. Perhaps most concerningly for health information professionals, intentionally fake diseases loaded into large language model’s training data are now cited as true conditions [36]. Because AI tools are correct in most instances, students and researchers alike can become accustomed to trusting the validity of their outputs, even if they know that these tools can facilitate fraud and make mistakes that are obvious to a human expert.

Yet despite these negative externalities, the scientific research community remains split on the role AI should have within research workflows. These opinions range from viewing AI as antithetical to the humanistic enterprise of genuine scientific inquiry [37], believing AI will transform every aspect of research [38], and everywhere in between [39]. Given these deltas, consensus among researchers on the appropriate roles for AI in research is unlikely. This creates another opportunity librarians and information scientists to take a leading role in guiding ethical adoption of AI.

## IMPLICATIONS FOR HEALTH SCIENCES LIBRARIANS

As domain experts in information systems, librarians and information professionals are poised to become leaders in how AI is implemented into research workflows. But to earn a space at the leadership table, librarians must be able to articulate clear guidance on AI use that is grounded in our broad conceptual knowledge about information architectures, our deep expertise in navigating information systems, and our own professional ethics. We suggest three criteria for information professionals to foreground during discussions over whether to use AI tools in research workflows: compliance, verifiability, and disclosure. Though not comprehensive of all ethical considerations, the Compliance, Verifiability, and Disclosure (CVD) Framework provides pragmatic, domain-agnostic guidance for weighing whether and when to use AI tools within research. These criteria are summarized in Table 1.

**Table 1:** The Compliance, Verifiability, and Disclosure (CVD) Framework for AI use in research workflows

Criteria	Guiding Question	Professional Implication
Compliance	Are you allowed to use AI for this task?	Rely on forward guidance from relevant ethics groups (e.g., Institutional Review Board) or prospective publication outlets (e.g., journal policies).
Verifiability	Are you capable of confirming the validity of an AI's output for this task?	Do not use AI tools to perform tasks that you do not know how to do yourself. And have verified the veracity of the output.
Disclosure	Are you prepared to describe (in detail) how you used AI during your research?	Only use AI for tasks that you would be willing to disclose to a counterparty (e.g., stakeholder, supervisor, journal editorial staff), and be prepared to describe exactly how AI was used.

*Compliance* recognizes that practical or legal constraints limit tasks that AI can be used for. Local institutional review boards, and journal editorial staffs, have wide ranging policies that span from extremely restrictive to very permissive. Prior to using AI, researchers should ascertain whether their data or other materials are permitted to be uploaded into a large language model in the first place.

*Verifiability* recognizes that a researcher’s prior knowledge and existing expertise should dictate what tasks an AI performs within a research process. This consideration suggests that researchers should only use AI tools to complete tasks they already know how to do. If a researcher cannot perform a given task using other means, then they cannot be confident in the validity of the AI’s output. Moreover, verifiability requires researchers to critically evaluate AI outputs against trusted, gold standard sources of evidence in addition to relying upon their own expertise [40].

*Disclosure* recognizes that researchers have an intuitive, personal sense of what tasks an AI should or should not perform on their behalf. If a researcher feels hesitant to acknowledge their use of AI to perform a task, it indicates their own recognition that they ought not to have used AI to do it.

## GUIDANCE FOR PROSPECTIVE *JMLA* AUTHORS

We remain skeptical of unrestricted use of AI tools within research. The *JMLA* editorial team wants its authors to grow and learn through process of conducting practitioner-based research. We see every task offloaded to an AI as a lost opportunity to potentially learn something new. To that end, we view writing as a unique and valuable form of thinking [41], through which a researcher decides which information to share with readers and how best to communicate it. Engaging deeply in the writing process is an essential step for understanding your own findings and for identifying which components of your work are worth highlighting. Authors who elect not to use AI when writing their manuscripts will likely have a richer understanding of their own work and may be better equipped to respond to reviewers’ comments and critiques.

For authors who wish to use AI tools as part of their workflows, we encourage *JMLA* authors to carefully consider the CVD Framework described in Table 1. Consult your local institutional review board prior to instigating a study. Be careful about offloading tasks to an AI that you do not already know yourself. Be prepared to proactively disclose, in detail, the full extent of how AI was used during your study planning, data collection, data analysis, and manuscript preparation.

Authors that elect to use AI must ensure that, as the human authors assuming responsibility for the content of their manuscript, they retain authorial control of their research outputs. Table 2 offers a comparison of Not Recommend Practices and Recommended Practices. While opinions on these specific examples will vary, we believe these are emblematic of our broader belief that research within health information science is a human-centered process: performed by humans for the benefit of humans.

**Table 2:** Possible approaches to using AI within manuscript preparation. Not Recommended Practices depict an author ceding excessive authorial control to an AI, while the Recommended Practices depict authors remaining in control of the writing process.

Manuscript Section	Not Recommended Practices	Recommended Practices
Abstract	“I have attached my draft manuscript. Please write me a structured abstract that is fewer than 250 words.”	“My draft abstract is below. I am currently at 260 words and need to get down to 250 words. Can you identify any words I could remove without altering the meaning of the section?”
Introduction	“I have attached a draft Methods and Results section for my paper. Please find me relevant citations from the literature and then use those citations to craft a five-paragraph Introduction section that justifies my study.”	“Below is a draft of my Introduction section. Does each paragraph have a comprehensive topic sentence that explains what the remainder of the paragraph will discuss?”
Methods	“I have uploaded a copy of my IRB application. Use the protocol within the document to write several paragraphs of prose for my Methods section.”	“Below is a draft of my Methods section. I have attempted to write the section chronologically. Are there any sections that appear out of order, or that may be unclear to a reader?
Results	“Run some inferential statistics on these data and report any significant findings.”	“I am planning to visualize these data using a vertical bar chart. Are there any other chart types that I could consider using?
Discussion	“What is interesting about my data?”	“What are some of the limitations of my research design that could be improved upon in future work?”

For *JMLA* authors who plan to use AI tools as their research assistants, copyeditors, and internal reviewers, we close with one final note to consider: large language models lack an analytic, human mind [42]. While AI tools invite anthropomorphism through their use of natural language and their impressive capabilities across domains [42], large language models cannot read [44]. Their ability to use word sequences to detect patterns within text is remarkable, but it is categorically a different process than how humans learn to read [45]. Likewise, although large language models can rapidly produce volumes of text, the language model has no understanding of what these words mean; the text they create is best described as what Shakespeare’s King Claudius called “words without thoughts” [46]. Though AI can produce literary text rich with simile and metaphor [47], the sui generis figures of speech created by AI lack the grounded human meaning that made The Bard’s dialogue so inimitable [48].

Conducting original research is an opportunity for librarians to make their mark on the research record, and to make the case that humans are central to the work of information and data. Researchers who offload their brainstorming, hypothesizing, and interpreting onto a large language model are taking ideas from a machine that cannot think; authors who offload their editing process onto a large language model are taking advice from a machine that can neither read nor write. We urge caution against removing ourselves out of these processes entirely.
